# Investigation of *Epilobium hirsutum* L. Optimized Extract’s Anti-Inflammatory and Antitumor Potential

**DOI:** 10.3390/plants13020198

**Published:** 2024-01-11

**Authors:** Ana-Maria Vlase, Anca Toiu, Octavia Gligor, Dana Muntean, Tibor Casian, Laurian Vlase, Adriana Filip, Ioana Bȃldea, Simona Clichici, Nicoleta Decea, Remus Moldovan, Vlad-Alexandru Toma, Piroska Virag, Gianina Crișan

**Affiliations:** 1 Department of Pharmaceutical Botany, Faculty of Pharmacy, Iuliu Hațieganu University of Medicine and Pharmacy, 8 Victor Babeș Street, 400012 Cluj-Napoca, Romania; gheldiu.ana@umfcluj.ro (A.-M.V.); octavia.gligor@gmail.com (O.G.); gcrisan@umfcluj.ro (G.C.); 2 Department of Pharmacognosy, Faculty of Pharmacy, Iuliu Hațieganu University of Medicine and Pharmacy, 8 Victor Babeș Street, 400012 Cluj-Napoca, Romania; atoiu@umfcluj.ro; 3 Department of Pharmaceutical Technology and Biopharmacy, Faculty of Pharmacy, Iuliu Hațieganu University of Medicine and Pharmacy, 8 Victor Babeș Street, 400012 Cluj-Napoca, Romania; dana.muntean@umfcluj.ro (D.M.); casian.tibor@umfcluj.ro (T.C.); 4 Department of Physiology, Faculty of Medicine, Iuliu Hațieganu University of Medicine and Pharmacy, 8 Victor Babeș Street, 400012 Cluj-Napoca, Romania; adrianafilip33@yahoo.com (A.F.); sclichici@umfcluj.ro (S.C.); nicoleta_decea@yahoo.com (N.D.); remus_ri@yahoo.com (R.M.); 5 Department of Molecular Biology and Biotechnology, Faculty of Biology and Geology, Babeș-Bolyai University, 44 Republicii Street, 400015 Cluj-Napoca, Romania; vlad.al.toma@gmail.com; 6 Institute of Biological Research, Branch of NIRDBS, 48 Republicii Street, 400015 Cluj-Napoca, Romania; 7 Department of Radiobiology and Tumor Biology, The Oncology Institute “Prof. Dr. Ion Chiricuță”, 34-36 Republicii Street, 400015 Cluj-Napoca, Romania; vpiroska@yahoo.com

**Keywords:** *Epilobium hirsutum*, optimized extract, acute rat paw inflammation, antioxidant potential, anti-inflammatory activity, Western Blot analysis

## Abstract

*Epilobium hirsutum* L., commonly known as hairy willowherb, is a perennial herbaceous plant native to Europe and Asia. In Romania, the *Epilobium* genus includes 17 species that are used in folk medicine for various purposes. This study aimed to investigate the anti-inflammatory and antitumor potential of the optimized extract of *Epilobium hirsutum* (EH) in animal models. The first study investigated the anti-inflammatory properties of EH optimized extract and the model used was carrageenan-induced paw inflammation. Wistar rats were divided into three groups: negative control, positive control treated with indomethacin, and a group treated with the extract. Oxidative stress markers, cytokine levels, and protein expressions were assessed. The extract demonstrated anti-inflammatory properties comparable to those of the control group. In the second study, the antitumor effects of the extract were assessed using the tumor model of Ehrlich ascites carcinoma. Swiss albino mice with Ehrlich ascites were divided into four groups: negative, positive treated with cyclophosphamide (Cph), Group 3 treated with Cph and EH optimized extract, and Group 4 treated with extract alone. Samples from the ascites fluid, liver, and heart were analyzed to evaluate oxidative stress, inflammation, and cancer markers. The extract showed a reduction in tumor-associated inflammation and oxidative stress. Overall, the EH optimized extract exhibited promising anti-inflammatory and antitumor effects in the animal models studied. These findings suggest its potential as a natural adjuvant therapeutic agent for addressing inflammation and oxidative stress induced by different pathologies.

## 1. Introduction

Medicinal plants are globally recognized as abundant sources of bioactive compounds that hold substantial therapeutic potential [1,2,3,4,5,6]. Recent advancements in biochemistry, chemistry, and medicine have enabled the identification of novel natural products with valuable therapeutic applications [7,8,9,10]. Consequently, numerous compounds derived from plants underwent clinical trials to assess their cytotoxic potential in cancer treatments, their effectiveness in addressing cardiovascular and metabolic diseases, their utility in managing inflammatory and related disorders, and their role as antimicrobial and antifungal agents [11,12,13,14,15,16].

In the pharmaceutical science field, the phytochemical characterization of medicinal plants takes precedence. As a result, several investigations have been initiated to establish scientific-evidence-backed rational herbal therapies [17,18]. To ensure the effectiveness, consistency, and safe usage of traditional herbal remedies, the quality control of medicinal plant extracts and the identification of their active constituents represent essential steps in the development of properly standardized and accessible herbal medicines [19,20,21,22,23,24].

The *Epilobium* genus, belonging to the *Onagraceae* family, presents a global distribution comprising more than 200 species, including 17 native to Romania. These plants are erect perennial herbs and often exhibit early flowering, sometimes within the first year [25,26]. Knowledge concerning indigenous *Epilobium* species remains incomplete and insufficient. This gap is particularly noteworthy given the abundance of these traditionally used medicinal plants within Romania’s native flora [27]. Considering that even minor variations in chemical composition can lead to significant disparities in bioactivity [20,21,28], the limited availability of scientifically supported data regarding Romania’s native flora poses a substantial limitation. 

Recent years have witnessed a growing interest in the phytochemistry of *Epilobium* plants, commonly known as willow herbs [27,29,30], supported by recent discoveries highlighting their beneficial effects in various health conditions, most notably in the prevention and treatment of prostate ailments [31,32]. Traditional folk medicine practices have long used the properties of willow herb infusions in managing benign prostate hyperplasia (BPH), prostatitis, as well as disorders of the bladder, kidneys, and urinary tract [33]. Moreover, their remarkable astringent, demulcent, and emollient properties have made them valuable remedies in addressing gastrointestinal issues, including diarrhea, dysentery, and other bowel and intestinal disorders associated with infection, inflammation, and irritation [34]. Notably, species such as *E. hirsutum*, *E. tetragonum*, *E. palustre*, and *E. angustifolium* have demonstrated significant antidiarrheal effects, achieved through the inhibition of muscular contractility and mobility [35]. These versatile plants have also found topical applications in treating various skin and mucosal conditions [36,37,38]. 

*Epilobium* species’ plant materials are abundant in secondary metabolites, especially polyphenols and lipophilic substances with therapeutic significance [26,27,34]. These include sterols, such as ergosterol, beta-sitosterol, and campesterol, as well as tocopherols, (α-, γ-, and δ-tocopherol) [27,34,39]. Furthermore, a recent study has highlighted the presence of various sterolic compounds in *E. hirsutum*, *E. parviflorum*, *E. palustre*, *E. angustifolium*, and *E. dodonaei* [27]. Additionally, some phenolic acid derivatives have been identified, while tannins and related compounds have been found in substantial quantities [27,30]. 

The phytcomplex of *Epilobium* species comprises a variety of bioactive constituents acknowledged for their anti-inflammatory and antitumor properties, including compounds like myricetin [40,41,42], hyperoside [43,44,45], quercitrin [46,47], kaempferol [48,49], gallic acid [50,51,52,53], catechin [54,55], and beta-sitosterol [56,57], among others. The synergistic effects of these compounds when present in total extracts play a significant role in enhancing biological activity. This aspect is crucial as isolated compounds may not exhibit the same level of bioactivity as when they are part of a complex mixture [26,34,58].

In the *Epilobium* genus, the cyclic dimeric ellagitannin oenothein B is a major compound, playing a key role in the various bioactivities attributed to the extracts of these species [27,31,59,60,61]. Numerous studies have highlighted the variability of oenothein B concentrations, with values ranging widely due to factors like species, plant part, and extraction techniques [39]. For instance, Bazylko et al. (2007) found 152.46 ± 4.92 mg/g in the aqueous extract of *E. angustifolium* [62], while Baert et al. (2015) identified the highest concentrations in *E. augustifolium* flowers (80 mg/g dry mass), followed by leaves (60 mg/g dry mass) extracted in acetone/water solution (4:1, *v*/*v*) [63]. On the other hand, Granica et al. (2012) achieved 72.91 ± 1.38 mg/g through ultrasonic extraction in *E. hirsutum* [61], Stolarczyk et al. (2013) noted the highest amounts in aqueous extracts of *E. hirsutum* (23.5 ± 0.3%) and *E. parviflorum* (22.7 ± 0.4%) [60], and Kiss et al. (2011) reported 333.6 ± 24.8 mg/g d.w. in aqueous extracts of *E. hirsutum* [64]. Moreover, Vlase et al. (2023) documented variability in oenothein B concentrations among *Epilobium* species. Their study found concentrations ranging from 41.88 ± 2.91 mg/g dry weight in 30% ethanol-water extracts of *E. palustre* aerial parts to 106.82 ± 7.45 mg/g dry weight in *E. dodonaei* using the same extraction solvent, highlighting the diversity of oenothein B content within the genus [27]. 

However, it is important to note that commercially available willow herb products often consist of mixtures of several species, each with distinct chemical compositions. These products are now commercially accessible in every EU member state, with growing applications in complementary therapies for BPH [29,31,32,33,65]. This variability raises questions about their biological equivalence. Therefore, there is an increasing need for standardized, evidence-based approaches to develop pharmaceutical preparations using these natural products. 

This study aimed to evaluate the anti-inflammatory and antitumor properties of phytocomplex obtained from the less-explored *E. hirsutum*. The extraction process was designed to maximize the content of phenolic compounds, known for their numerous health benefits, as detailed in a previous publication [27]. The current investigation employed two distinct animal models to provide an in-depth assessment of the biological effects of the optimized *E. hirsutum* extract. 

## 2. Results

The proinflammatory enzyme cyclooxygenase-2 (COX2) [66] was statistically significantly decreased in the groups treated with *E. hirsutum* optimized extract (EH) (*p* < 0.0001 vs. the control—the group treated with carboxymethyl cellulose, CMC) and Indomethacin (IND) (*p* < 0.0001 vs. CMC)—with significant differences between these two groups. This result shows a powerful inhibition of the COX-2 inflammation pathway exerted by the EH extract. The nuclear factor erythroid 2–related factor 2 (NRF2), a protein marker of oxidative stress response [67], was decreased by IND and increased in the group treated with EH extract (*p* < 0.05 vs. CMC), and the difference between the two treated groups was statistically significant (*p* < 0.01). These results indicate that IND had induced a lower expression of NRF2 compared to CMC, while EH treatment significantly increased NRF2 expression compared to both CMC and IND, which could suggest a protective antioxidant effect of the EH extract.

**Figure 1:**
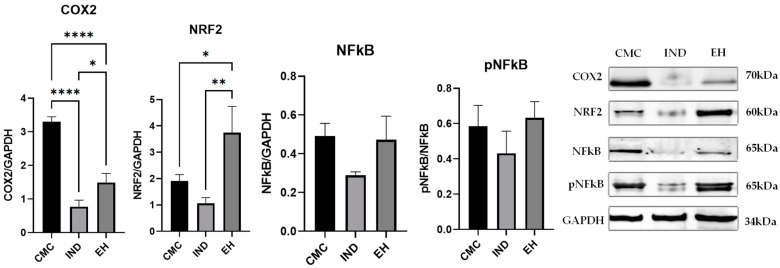
Quantification of cyclooxygenase-2 (COX2), nuclear factor erythroid 2–related factor 2 (NRF2), nuclear factor kappa B (NFκB), and its phosphorylated form (pNFκB) expression in the rat paw tissue at 2 h after inducing local inflammation (CMC—negative control treated with carboxymethyl cellulose; IND—positive control treated with indomethacin; EH—group treated with *E. hirsutum* optimized extract). Western blot was used for the respective analysis; results were normalized to glyceraldehyde 3-phosphate dehydrogenase (GAPDH) as internal standard. Statistical analysis was performed using one-way ANOVA test with Tukey’s multiple comparison post-hoc test. Values are presented as mean ± SD (* *p* < 0.05, ** *p* < 0.01, **** *p* < 0.0001).

Nuclear factor kappa B (NFκB) pathway activation was increased by EH, mainly through the activation of the existent NFκB protein (pNFκB). NFκB activation and the augmented NRF2 led to a robust antioxidant and anti-inflammatory effect. By contrast, the IND-treated group showed decreased levels of NFκB activation, but without significance, as compared to controls and the EH-treated group (Figure 1). 

**Figure 2:**
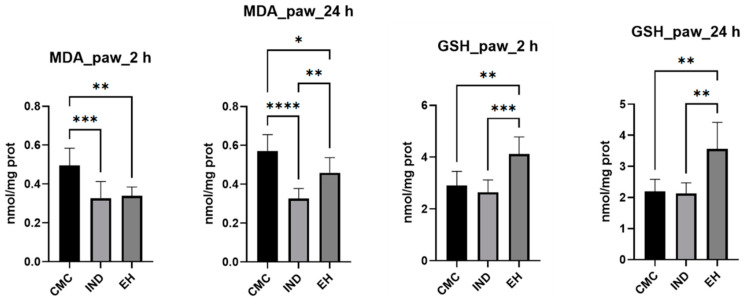

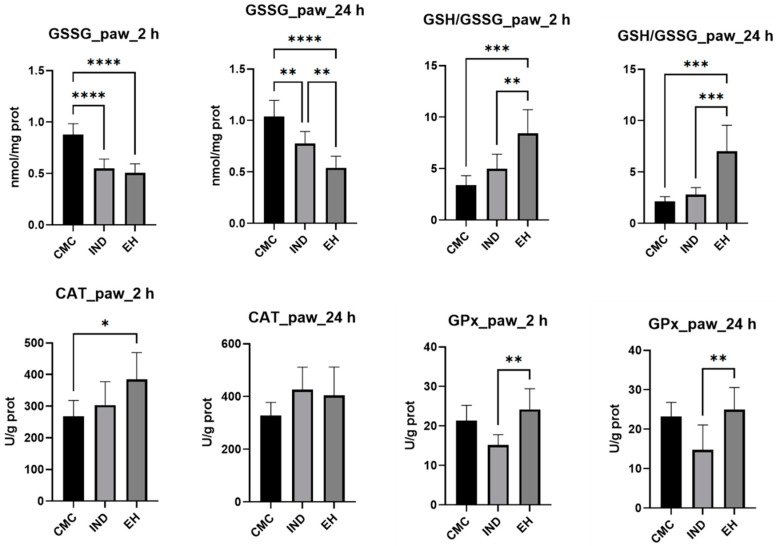
Levels of oxidative stress markers: malondialdehyde (MDA), reduced glutathione (GSH), oxidized glutathione (GSSG), and their ratio (GSG/GSSG), catalase (CAT), and glutathione peroxidase (GPx) activities from the rats paw tissue homogenates (sampled at 2 and 24 h after inflammation) following a 4-day treatment with indomethacin (IND) and *E. hirsutum* optimized extract (EH), respectively. Carboxymethyl cellulose (CMC)—negative control. Values are presented as mean ± SD. Statistical analysis was performed using one-way ANOVA, with Tukey’s multiple comparisons post-hoc test (* *p* < 0.05 vs. control group, ** *p* < 0.01 vs. control group, *** *p* < 0.0001 vs. control group, **** *p* < 0.00001 vs. control group).

Moreover, the rat paw tissue was collected at 2 and 24 h to capture both the acute phase and the beginning of the resolution phase of the inflammatory process [68,69]. Oxidative stress parameters were measured, such as the lipid peroxidation indicator, malondialdehyde (MDA), non-enzymatic endogenous antioxidants, including reduced glutathione (referred to as GSH) and oxidized glutathione (referred to as GSSG), and the ratio of GSH to GSSG. Additionally, the activity of enzymatic antioxidants, specifically catalase (CAT) and glutathione peroxidase (GPx), was assessed to investigate the reduction in oxidative stress in homogenized samples of rat paw tissue (Figure 2). 

**Figure 3:**
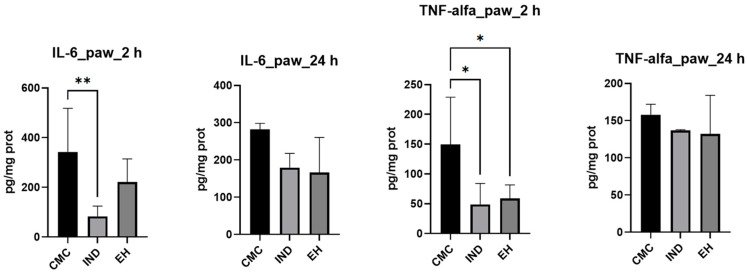
Proinflammatory cytokines levels (IL-6—interleukin-6; TNF-alfa—tumor necrosis factor-alfa) within the rat paw tissue homogenates samples after a 4-day treatment with indomethacin (IND) and *E. hirsutum* optimized extract (EH), respectively. Carboxymethyl cellulose (CMC)—negative control. Values are given as mean ± SD. Statistical analysis was performed using one-way ANOVA, with Tukey’s multiple comparisons post-hoc test (* *p* < 0.05 vs. control group, ** *p* < 0.01).

**Figure 4:**
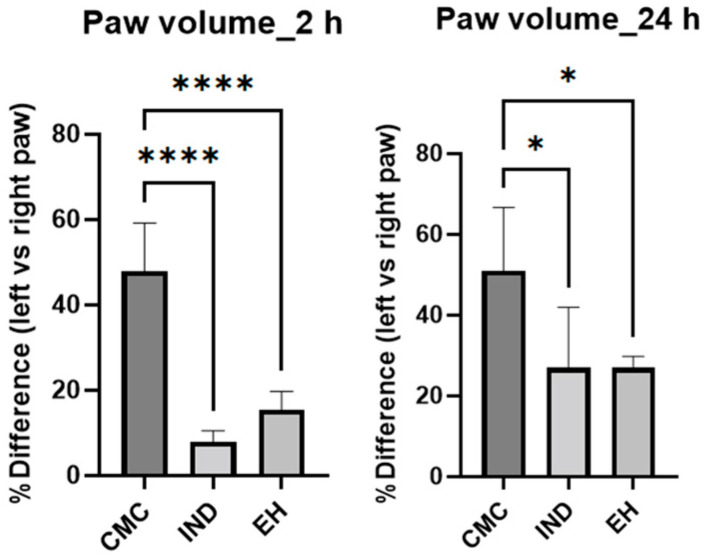
The percentage difference between left rat paw volume (negative control) versus right rat paw volume (positive control) determined with a plethysmometer, at 2 and 24 h after carrageenan administration. CMC—animals treated with carboxymethyl cellulose (negative control); IND—rats treated with indomethacin (positive control); EH—animals treated with *E. hirsutum* optimized extract (test group) (* *p* < 0.05 vs. control group, **** *p* < 0.00001 vs. control group).

To provide a comprehensive view of the inflammatory process, the cytokine levels IL-6 and TNF-alfa were measured (Figure 3) in dynamics at 2 and 24 h after inducing inflammation [70]. Additionally, the variation in paw volume between the negative control, positive control, and experimental group was examined, expressing the results as a percentage relative to the difference between the left paw before and after carrageenan administration and the right paw, treated with CMC, IND, or EH, respectively (Figure 4). 

At 2 h post carrageenan administration, both the IND and the EH groups displayed a statistically significant decrease in paw volume, highlighting a potent anti-inflammatory action (Figure 4). At 24 h observation, the diminution in paw volume remained statistically significant for both treatments, suggesting the persistent or possibly prolonged anti-inflammatory potency of the EH extract.

**Figure 5:**
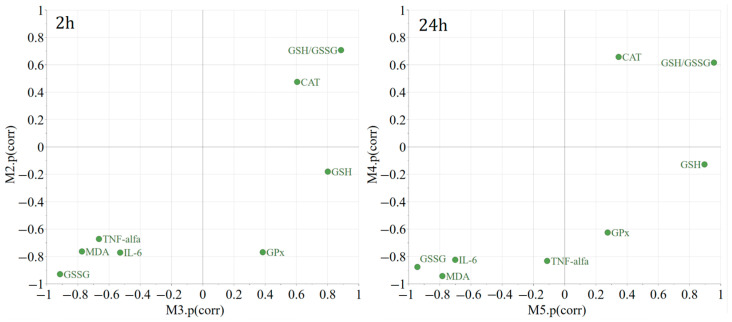
M2 (2 h)—Indomethacin (IND—positive control) vs. carboxymethyl cellulose (CMC—negative control); M3 (2 h)—*E. hirsutum* optimized extract vs. CMC (negative control); M4 (24 h)—IND (positive control) vs. CMC (negative control); M5 (24 h)—*E. hirsutum* optimized extract vs. CMC (negative control).

Orthogonal Projections to Latent Structures Discriminant Analysis (OPLS-DA) was utilized to explore the distinct expression patterns of the chosen variables or biological parameters as a result of the treatments in comparison to the negative control. To facilitate the visual differentiation and identification of commonalities among treatments, Shared and Unique Structures (SUS) plots were created. These plots display the modeled correlation vectors (p[corr]) from two distinct OPLS-DA models against each other (Figure 5). Two hours following the induction of inflammation, compared to the negative control, both the positive control treatment and the EH treatment led to a significant reduction in GSSG and MDA, with a more pronounced decrease in MDA levels. Compared to the negative control, both the IND and the EH treatment resulted in an increase in the GSH/GSSG ratio (the change is statistically significant only for EH treatment versus the CMC group). For unique effects, the EH treatment resulted in a statistically significant increase in GSH, whereas the positive control treatment did not produce this effect. The EH treatment seems to be effective in modulating oxidative stress markers by significantly increasing the GSH levels and the GSH/GSSG ratio compared to the negative control. These findings suggest that the EH extract has potent antioxidant properties. The EH treatment led to a statistically significant increase in catalase (CAT) activity at 2 h. The IND treatment did decrease GPx significantly, which is not observed with the EH treatment, indicating a differential effect on this enzyme’s activity. The reduction in TNF-alpha and IL-6 for both the IND and EH groups was observed at 2 h, being statistically significant regarding IL-6 for the IND group, and for both the IND and EH groups considering TNF-alfa. The lack of statistical significance in the alteration in TNF-alpha and IL-6 levels at 24 h suggests that while there may be a trend towards anti-inflammatory effects at the beginning (2 h), the data at this time point do not provide strong evidence of a significant impact on these inflammatory markers, as the inflammation naturally subsides. This comparative analysis highlights that the temporal aspect of the IND and EH extract effects is crucial. While both treatments appear to modulate inflammatory and oxidative stress markers, their specific effects can differ significantly over time, which is an important consideration for their therapeutic use and potential side effects. 

**Figure 6:**
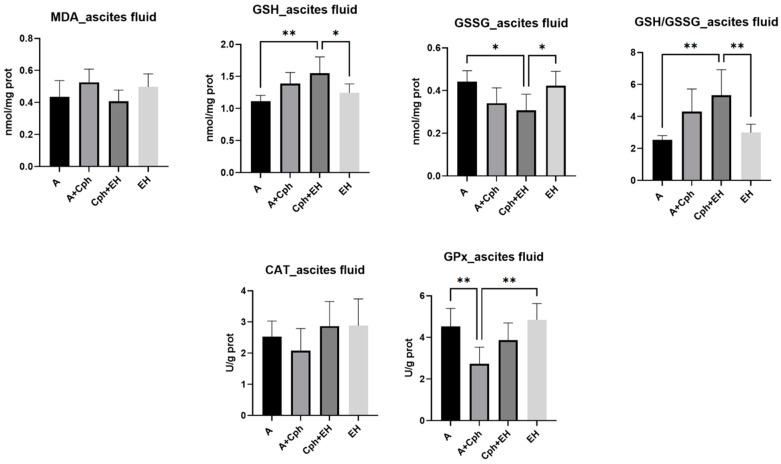
Levels of oxidative stress markers: malondialdehyde (MDA), reduced glutathione (GSH), oxidized glutathione (GSSG), and their ratio (GSG/GSSG), catalase (CAT), and glutathione peroxidase (GPx) activities from the mice ascites samples after a 10-day treatment with cyclophosphamide (Cph), association of Cph and *E. hirsutum* optimized extract (EH), and EH extract alone, respectively. Values are presented as mean ± SD. Statistical analysis was performed using one-way ANOVA, with Tukey’s multiple comparisons post-hoc test (* *p* < 0.05 vs. control group, ** *p* < 0.01 vs. control group).

For the second study, performed on mice with Ehrlich ascites, an assessment of the lipid peroxidation marker malondialdehyde (MDA) was performed, alongside non-enzymatic endogenous antioxidants (reduced glutathione noted GSH, oxidated glutathione noted GSSG, and their ratio GSH/GSSG), as well as enzymatic antioxidants (catalase (CAT) and glutathione peroxidase (GPx), to examine oxidative stress reduction in ascites fluid samples. The results are further displayed in Figure 6. The analysis of ascites fluid provided insight into the tumor microenvironment, the host’s immune response, and the efficacy of therapeutic interventions. As such, lipid peroxidation was influenced by the therapeutic association of cyclophosphamide and EH extract (Cph + EH), with levels comparable with those of the negative control group; yet, no statistical significance was present in this case. The levels of endogenous non-enzymatic antioxidants were elevated after treatment with the therapeutic association, namely GSH (*p* < 0.01 vs. untreated group). EH alone influenced only the GSSG levels (*p* < 0.05 vs. Cph + EH group). The cotreatment of Cph + EH also led to an increase in the GSH/GSSG ratio (*p* < 0.01 vs. untreated group and EH group). CAT activity was increased by both the EH treatment alone and by its association with Cph, especially in comparison with the Cph group. Although these results did not hold statistical significance. GPx activity was enhanced as well for both therapeutic approaches; in this case, the results were statistically significant (*p* < 0.01 vs. Cph group).

**Figure 7:**
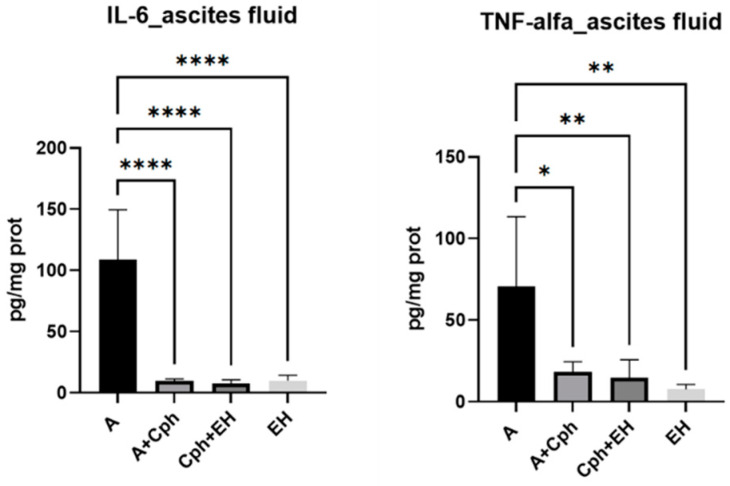
Proinflammatory cytokines amount (IL-6—interleukin-6, TNF-alfa—tumor necrosis factor-alfa) within the mice ascites samples after a 10-day treatment with cyclophosphamide (Cph), association of Cph and *E. hirsutum* optimized extract (EH), and EH extract alone, respectively. Values are depicted as mean ± SD. Statistical analysis was performed using one-way ANOVA, with Tukey’s multiple comparisons post-hoc test (* *p* < 0.05 vs. control group, ** *p* < 0.01, **** *p* < 0.0001 vs. control group).

The levels of IL-6 and TNF-α in the ascitic fluid samples were quantified (as depicted in Figure 7). There was a statistically significant decrease in IL-6 concentrations across all experimental groups, including the Cph group and those treated with EH, with *p*-values of less than 0.0001 when compared to the untreated group. Similarly, TNF-α levels were significantly altered in all test groups, with *p*-values of less than 0.01 observed in both the Cph + EH and EH groups, and a *p*-value of less than 0.05 noted for the Cph group alone. 

**Figure 8:**
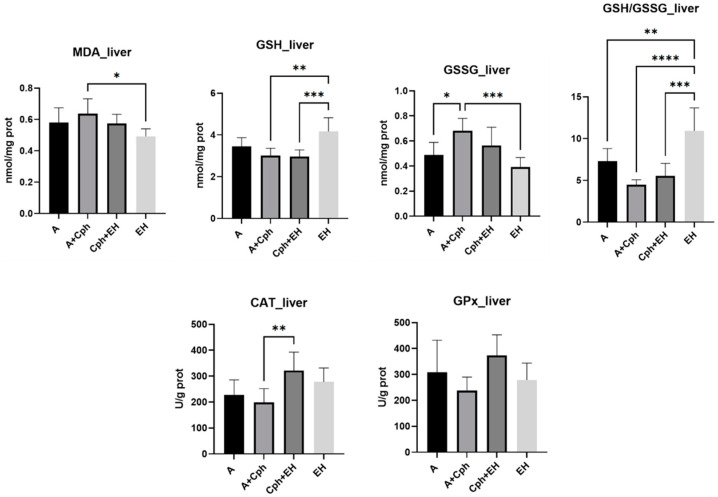
Levels of oxidative stress markers: malondialdehyde (MDA), reduced glutathione (GSH), oxidized glutathione (GSSG), and their ratio (GSG/GSSG), catalase (CAT), and glutathione peroxidase (GPx) activities from the mice liver samples after a 10-day treatment with cyclophosphamide (Cph), association of Cph and *E. hirsutum* optimized extract (EH), and EH extract alone, respectively. Values are presented as mean ± SD. Statistical analysis was performed using one-way ANOVA, with Tukey’s multiple comparisons post-hoc test (* *p* < 0.05, ** *p* < 0.01, *** *p* < 0.001, **** *p* < 0.0001).

The liver is a common site for metastasis in many types of cancers, including Ehrlich ascites carcinoma. It is also a critical organ for drug and bioactive compound metabolism and can be affected by both the tumor burden and the toxicity of therapeutic agents. Examining liver tissue helped assess the impact of cancer and treatment on hepatic function and architecture. Therefore, oxidative stress marker levels were also determined within the obtained liver tissue samples (Figure 8). EH treatment alone induced a decrease in MDA levels in a statistically significant manner in the experimental mice (*p* < 0.05) compared to the animals receiving Cph. The increase in non-enzymatic antioxidants’ concentration was noticeable, as EH treatment alone influenced GSH levels and the GSH/GSSG ratios significantly (*p* < 0.001 vs. Cph + EH group and *p* < 0.0001 vs. Cph group, respectively). GSSG levels were only statistically increased through the Cph treatment (*p* < 0.001). The activity of enzymatic antioxidants was also observed to increase; however, in this case, for the Cph + EH-treated group, there was statistical significance for CAT (*p* < 0.01 vs. Cph group).

**Figure 9:**
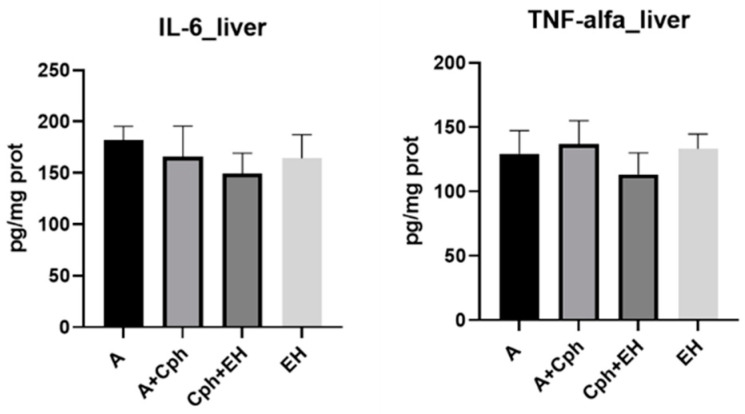
Proinflammatory cytokines levels (IL-6—interleukin-6, TNF-alfa—tumor necrosis factor-alfa) within the mice liver samples after a 10-day treatment with cyclophosphamide (Cph), association of Cph and *E. hirsutum* optimized extract (EH), and EH extract alone, respectively. Values are depicted as mean ± SD. Statistical analysis was performed using one-way ANOVA, with Tukey’s multiple comparisons post-hoc test.

In terms of inflammation markers, as seen in Figure 9, their respective concentrations remained elevated within the tested samples, although these results did not present statistical significance. 

**Figure 10:**
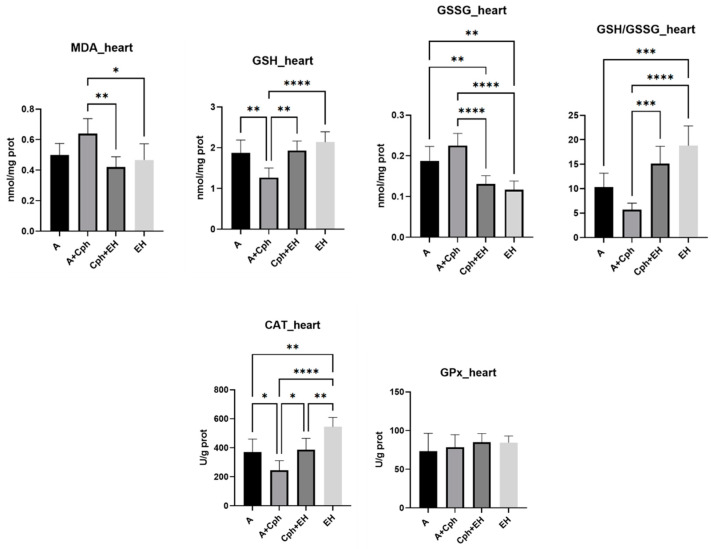
Levels of oxidative stress markers: malondialdehyde (MDA), reduced glutathione (GSH), oxidized glutathione (GSSG), and their ratio (GSG/GSSG), catalase (CAT), and glutathione peroxidase (GPx) activities from the mice heart samples after a 10-day treatment with cyclophosphamide (Cph), association of Cph and *E. hirsutum* optimized extract (EH), and EH extract alone, respectively. Values are presented as mean ± SD. Statistical analysis was performed using one-way ANOVA, with Tukey’s multiple comparisons post-hoc test (* *p* < 0.05, ** *p* < 0.01, *** *p* < 0.001, **** *p* < 0.0001).

While the heart is not a typical site of direct tumor invasion, systemic effects of cancer and adverse reactions to treatment can impact the cardiac tissue. Cardiotoxicity is an important side effect of many anticancer drugs; hence, monitoring heart tissue helps evaluate the cardioprotective or damaging effects of treatments. Thus, the same biological parameters were also evaluated in heart tissue samples isolated from the four animal test groups (Figure 10) and notable variations in their levels were also observable. MDA levels were statistically significantly decreased for the group receiving the co-treatment of Cph + EH in comparison to the Cph group (*p* < 0.01), as well as for the group receiving the treatment with EH alone (*p* < 0.05) in comparison to the Cph group. GSH levels were statistically significant influenced by the EH treatment (*p* < 0.0001), followed by therapeutic association with Cph (*p* < 0.01). On the other hand, the Cph treatment alone led to an increase in GSSG levels in comparison to the therapeutic association (*p* < 0.0001) and treatment with EH alone (*p* < 0.0001). The GSSG levels of the untreated group were also found to have been increased as opposed to those of both therapeutic approaches (*p* < 0.01). The GSH/GSSG ratio was consequently increased, mainly through the EH treatment, in comparison to all other experimental groups (*p* < 0.0001). The Cph + EH association managed to reach the second highest levels for the GSH/GSSG ratio against the Cph group (*p* < 0.001). EH also lead to a highly discernable increase in CAT activity (*p* < 0.0001 vs. Cph, *p* < 0.001 vs. Cph + EH and the untreated groups). The outcomes pertaining to glutathione peroxidase (GPx) did not exhibit significant variance.

**Figure 11:**
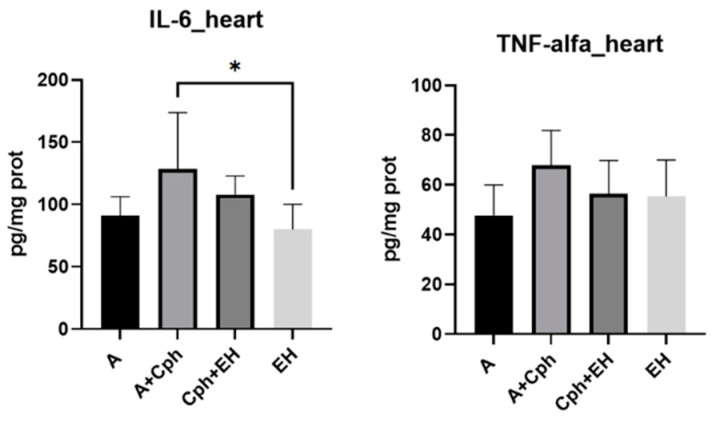
Proinflammatory cytokines amount (IL-6—interleukin-6; TNF-alfa—tumor necrosis factor-alfa) within the mice heart samples after a 10-day treatment with cyclophosphamide (Cph), association of Cph and *E. hirsutum* optimized extract (EH), and EH extract alone, respectively. Values are presented as mean ± SD. Statistical analysis was performed using one-way ANOVA, with Tukey’s multiple comparisons post-hoc test (* *p* < 0.05 vs. control group).

Figure 11 depicts the results concerning the levels of pro-inflammatory markers determined in heart tissue samples. The EH treatment led to a statistically significant decrease in IL-6 levels in contrast to the Cph treatment (*p* < 0.05).

**Figure 12:**
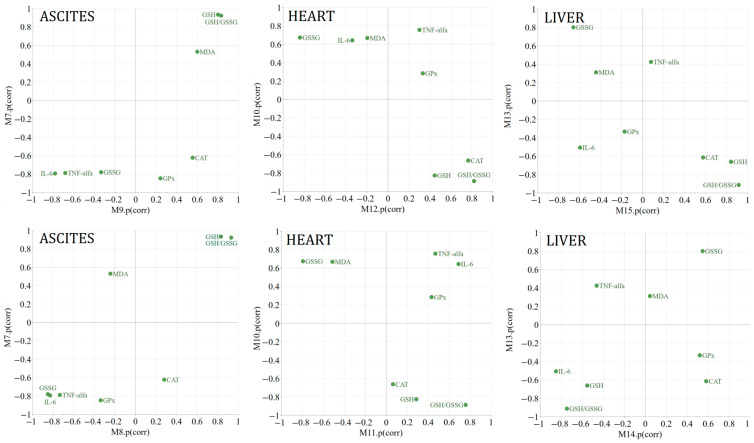
M7 (ascites fluid), M10 (heart), M13 (liver)—ascites + cyclophosphamide (positive control) vs. ascites (negative control); M9 (ascites fluid), M12 (heart), and M15 (liver)—*E. hirsutum* optimized extract vs. ascites (negative control); M8 (ascites fluid), M11 (heart), and M14 (liver)—cyclophosphamide + *E. hirsutum* optimized extract vs. ascites (negative control).

In the context of an Ehrlich ascites carcinoma mouse model, the therapeutic effects of Cph (positive control), an established chemotherapeutic agent, and an optimized extract of *Epilobium hirsutum* (EH) were evaluated through SUS plot analysis across three distinct biological matrices: ascites fluid, heart, and liver samples (Figure 12).

Ascites fluid analysis: Both treatments, Cph and EH, led to a common effect of decreasing IL-6 and TNF-alfa levels and increasing GSH and the GSH/GSSG ratio, indicating a shared anti-inflammatory and antioxidative mechanism. Unique to Cph treatment was a further decrease in GSSG, GPx, and CAT levels, whereas EH showed an increase in these variables compared to the negative control. Co-administration of EH with Cph resulted in significant changes for GSH, GSSG, and their ratio.

Heart analysis: The Cph treatment resulted in an increase in MDA, IL-6 and TNF-alfa and a decrease in GSH and the GSH/GSSG ratio. The EH treatment, on the other hand, was associated with a decrease in MDA, GSSG levels and an increase in CAT activity and the GSH/GSSG ratio, suggesting a cardioprotective antioxidative effect. Co-administration of EH with Cph led to changes as well, reducing the GSSG and elevating the CAT. The impact on the GSH/GSSG ratio remained consistent with the EH treatment.

Liver analysis: Significant changes were identified for the variables GSH, GSSG, and their ratio. The treatment with Cph led to an increase in GSSG levels and a concurrent decrease in GSH levels and the GSH/GSSG ratio, indicating an oxidative stress. Conversely, the treatment with EH exhibited opposite effects, namely a decrease in GSSG levels and an increase in both GSH levels and the GSH/GSSG ratio, suggesting an enhancement in the antioxidative response. The co-administration of EH with Cph mirrored the effects found with Cph alone, with a statistically significant effect only observed for GSSG versus the negative control group.

These findings suggest that the EH treatment, both alone and in conjunction with Cph, influenced oxidative and antioxidative markers, with a notable impact on the antioxidative defense system, as evidenced by the enhanced GSH levels and CAT activity. These effects are consistent across different biological matrices, including ascites fluid and heart tissue, underscoring the potential of EH as an adjunct therapy to modulate oxidative stress in the context of cancer treatment. The data illustrate that while Cph exerts pronounced pro-oxidative and anti-inflammatory effects, the addition of EH appears to modulate this response, enhancing antioxidative defenses and potentially mitigating inflammatory responses. The consistent patterns observed across these biomarkers reflect the complex interplay in the context of Ehrlich ascites carcinoma between oxidative stress and the body’s response to cancer therapy, underscoring the potential benefits of integrating natural extracts like EH with conventional chemotherapeutic agents.

**Figure 13:**
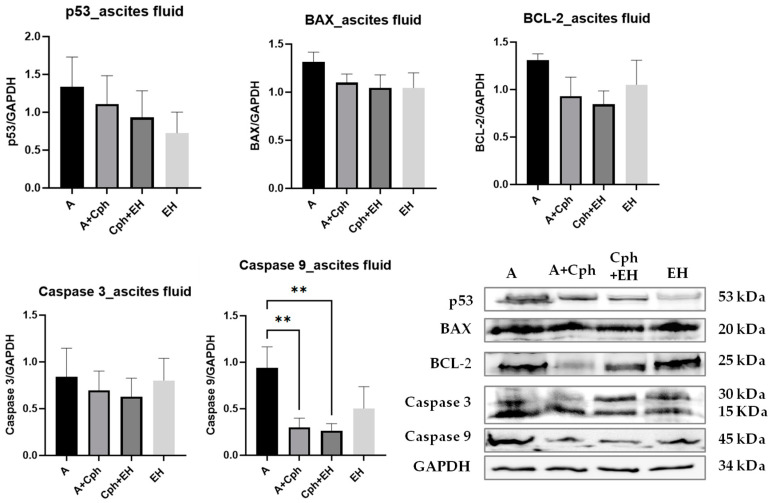
Caspase-3, caspase-9, tumor suppressor protein p53, B-cell lymphoma 2 (BCL-2), and BCL-2-like protein 4 (BAX) levels in mice ascites samples after 10-day treatment with cyclophosphamide (Cph), association of Cph and *E. hirsutum* optimized extract (EH), and EH extract alone, respectively. Western blot was used for the respective analysis; results were normalized to glyceraldehyde 3-phosphate dehydrogenase (GAPDH) as internal standard. Statistical analysis was performed using one-way ANOVA, with Tukey’s multiple comparisons post-hoc test. Values are given as means ± SD (** *p* < 0.01 vs. control group).

Figure 13 illustrates the modifications in proteins p53, BAX, BCL-2, caspase-3, and -9 from ascites fluid samples, along with their Western blot images. Caspase-3 level was decreased in ascites fluid samples after the Cph treatment and Cph + EH co-treatment. The EH treatment alone did not influence the caspase-3 level in comparison to the control group. Caspase-9 levels were significantly reduced in Cph and Cph + EH groups (*p* < 0.01) vs. control, without significant differences between the two groups. The EH treatment slightly reduced caspase-9 but lacked statistical significance. The p53 levels were decreased in comparison to control group and the most important decrease was observed in the EH group. The antiapoptotic protein BCL-2 level was decreased in the Cph and Cph + EH groups, while in the latter, the effect was stronger, though not statistically significantly compared to controls. In all treated groups, the BAX level was decreased compared to controls, although not significantly. Overall, the p53 apoptosis induction pathway was inhibited by exposure of the ascites cells to Cph and this effect was increased by EH. This is probably due to the antioxidant effect of the extract, which reduces the pro-oxidant environment of the cancer cells and can act as a scavenger for the free radicals induced by inflammation and chemotherapy. 

**Figure 14:**
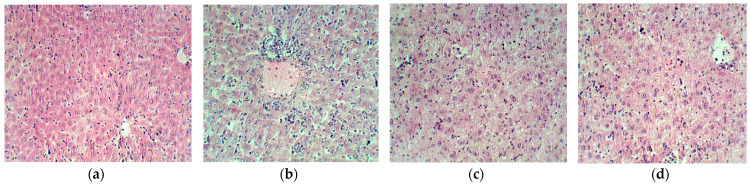
Histological appearance of the liver in the control group ((**a**)—ascites) and experimental groups ((**b**)—ascites + cyclophosphamide; (**c**)—cyclophosphamide + *E. hirsutum* optimized extract; (**d**)—*E. hirsutum* optimized extract). Hematoxylin–eosin staining, ×200.

As revealed by hematoxylin–eosin staining (Figure 14), the induction of ascites (a) resulted in typical but discrete histological changes manifested by the faint presence of tumor cells in the liver sinusoids. Cph administration reduced the population of neoplastic cells in the liver parenchyma (b) and this process was accelerated after Cph administration together with EH extract (c). The administration of EH extract (d) resulted in significant liver damage, but the tumor cell population was comparable to that in the control group.

**Figure 15:**
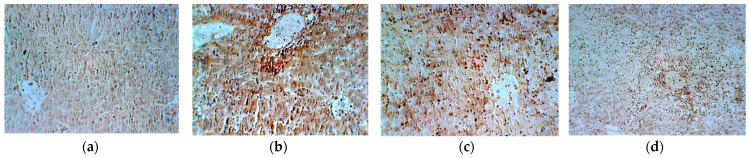
Histological appearance of the liver after reticulin staining in the control group ((**a**)—ascites) and experimental groups ((**b**)—ascites + cyclophosphamide; (**c**)—cyclophosphamide + *E. hirsutum* optimized extract; (**d**)—*E. hirsutum* optimized extract). ×200.

Reticulin dynamics (Figure 15) suggested necrosis processes with large areas of hepatocyte loss in Groups 1 (ascites) and 3 (Cph + EH). In contrast, in Groups 2 (ascites + Cph) and 4 (EH), the liver parenchyma was noticed as near normal. By highlighting these fibers, the staining helps assess the architecture of the hepatocytes and extracellular matrix (space of Disse), such as the expansion in regenerative and neoplastic conditions, compression of plates in nodular regenerative hyperplasia, and collapse of the reticulin framework in necrosis [71].

**Figure 16:**
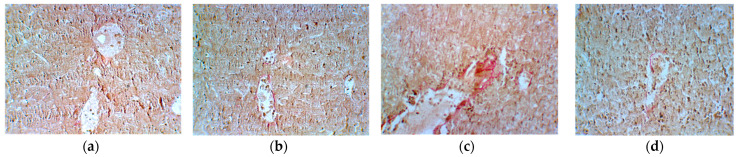
Histological appearance of the liver in the control group ((**a**)—ascites) and experimental groups ((**b**)—ascites + cyclophosphamide; (**c**)—cyclophosphamide + *E. hirsutum* optimized extract; (**d**)—*E. hirsutum* optimized extract) stained with Van Gieson method, ×200.

The Van Gieson histological staining technique is commonly used to visualize collagen fibers and elastic fibers in tissues. Collagen fibers appear red or pink, while elastic fibers appear yellow, brown, or black (Figure 16). This staining technique provides information about the presence, distribution, and quantity of collagen and elastic fibers in the tissue [72]. In this case, the Van Gieson method indicates small vascular wall deposits with collagen in Groups 2 (ascites + Cph) and 3 (Cph + EH).

**Figure 17:**
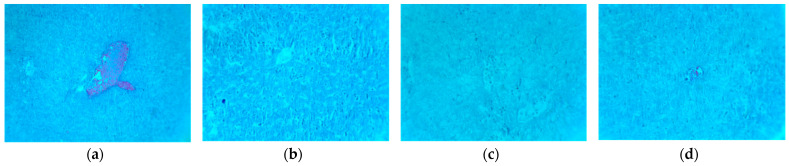
Histological appearance of the liver in the control group ((**a**)—ascites) and experimental groups ((**b**)—ascites + cyclophosphamide; (**c**)—cyclophosphamide + *E. hirsutum* optimized extract; (**d**)—*E. hirsutum* optimized extract) with Mallory’s trichrome staining.

The Mallory’s trichome staining procedure involves a series of steps that includes staining the tissue with Weigert’s hematoxylin to visualize nuclei, followed by three different dyes: acid fuchsin, aniline blue, and orange G. The acid fuchsin stains cytoplasm and muscle fibers red, aniline blue stains collagen blue, and orange G stains the background and erythrocytes orange [73]. Mallory staining has revealed no perivascular extension of the collagen with origin in the vascular wall (Figure 17).

The histological changes in the reticulin deposits and collagen infiltration were investigated in a semiquantitative manner, as evidenced by the reticulin and Van Gieson staining techniques, and are summarized in Table 1. 

## 3. Discussion

NRF2 is involved in the inflammation process by recruiting inflammatory cells as well as regulating the expression of pro-inflammatory genes, such as COX2 and iNOS [74]. When activated, NRF2 translocates to the nucleus and binds to Antioxidant Response Elements (ARE) in the promoter regions of various genes involved in antioxidant and cytoprotective responses [75,76]. This activation leads to the upregulation of several antioxidative enzymes and proteins, thereby enhancing the cell’s capacity to neutralize reactive oxygen species (ROS) and protect against oxidative damage [76,77]. The induction of NRF2 in our study suggests an enhanced cellular response to mitigate oxidative stress, which is a critical factor in the context of inflammation.

Oxidative stress leads to NFκB activation, which in turn, causes the upregulation of acute-phase-protein genes, cytokines, and interleukins [78]. The results showing an inhibitory effect of the EH treatment on COX2 levels were in accordance with another in vivo study involving testosterone propionate-induced benign prostatic hyperplasia in castrated Sprague Dawley rats, in which decreased COX2 expression and NFκB pathway down-regulation were noted after treatment with *n*-butanolic *Epilobium angustifolium* L. extracts compared to a positive control of finasteride [79]. The present results were also similar to those of an in vitro digestion study performed by Szwajgier et al., using aqueous *E. angustifolium* L. extracts obtained via ultrasound-assisted extraction [80]. In addition, an in vitro study using PC3 prostate cancer cells also attested the anti-inflammatory and antiproliferative effects of methanolic and hydromethanolic EH extracts. In a recent study conducted by Zengin et al., a reduced expression of COX2 and TNF-α genes was noted, and bioinformatics analyses indicated myricetin and oenothein B as the bioactive compounds behind these effects. While there was a reduction in IL-8 levels, an elevation in IL-6 was observed. This increase might be attributed to the complex role of IL-6, which can vary depending on the type of tissue involved and the specific conditions of the experiment. In addition, the study reported that the methanolic extracts of EH reduced NFκB gene expression, a finding that stands in contrast to the current results, where no change was observed [30]. Several other types of extracts of different *Epilobium* species, ranging from aqueous to dichloromethane, have been shown to positively influence inflammation, including moderately inhibiting NFκB, and increase the inhibition of COX2. The antiphlogistic and antioxidant effects of species pertaining to this genus were credited again to several natural compounds: ellagitannins such as oenothein B, as well as its various metabolites, such as urolithitins; and flavonoid compounds such as myricetin, and quercetin glycosides, for instance, quercetin glucuronide [26,34,58,64,81]. The optimized EH extract selected for this study reflected the findings from the scientific literature, as it was also noted to contain high levels of polyphenolic compounds, especially oenothein B (73.49 ± 3.89 mg/g d.w.) [27]. In our previous study, the selection of a 30% ethanol–water solvent for extracting polyphenols from EH was based on optimization using Design of Experiments (DoE) tools. Although this solvent mixture is less suitable for extracting apolar compounds like sterols and tocopherols, it was found to be efficient for phenolic compounds, notably oenothein B, which were the primary focus due to their recognized bioactive properties [27].

Lipid peroxidation is an important indicator of oxidative stress induced by reactive oxygen species (ROS), resulting in various compounds which affect cellular health. MDA is one of the main products of this cellular process, therefore constituting an important marker of oxidative stress [82]. GSH is an important non-enzymatic antioxidant involved in the protection against ROS, as well as redox control by scavenging oxygen-derived free radicals, through its conversion to GSSG. Endogenous antioxidant enzymes are responsible for metabolizing reactive species, as well as for upholding cellular redox homeostasis. Examples include CAT and GPx [83]. The decrease in MDA levels observed for liver and heart tissues for the EH-treated group are in agreement with other scientific results, albeit concerning an experimental seizure model in Swiss albino mice. In this case, treatment consisted of valproate alongside EH ethanolic extracts. CAT and GSH levels in the collected brain tissue were also improved after treatment [84]. This amelioration was also found in the case of the present study, namely for the ascites fluid, liver, and heart tissues in the experimental rat model. The positive influence over GPx activity is also in accordance with a previously cited in vitro study involving *E. angustifolium* L. [80]. Decreased MDA and GPx levels were also observed in rat prostate tissue samples after treatment with *n*-butanolic extracts of *Epilobium angustifolium* L. from a previously cited in vivo study [79]. Karakurt et al. also found that intraperitoneally administered aqueous EH extract led to an increased activity of chemopreventive enzymes such as GPx in Wistar rats. Moreover, EH injection was revealed to have increased the levels of mRNA expression of these particular enzymes [85].

The decreased IL-6 and TNF-α levels in ascites samples after treatment with EH alone and the association of Cph + EH were in accordance with the results of another previously cited in vivo study concerning castrated Sprague Dawley rats with benign prostatic hyperplasia. In that case, reduced IL levels, among which IL-6, were also reported. *n*-butanolic extracts of *Epilobium angustifolium* L. were administered as treatment [79]. Another in vivo study concerning the antihyperglycemic effect of *Epilobium parviflorum* Schreb. in Wistar rats with induced type 2 diabetes mellitus reported that TNF-α serum levels decreased after co-treatment with pioglitazone and aqueous leaf extract of *Epilobium parviflorum* Schreb. TNF-α was also responsible for influencing insulin sensitivity, the modulation of free fatty acids in plasma, and lipid synthesis. The treatment also exhibited anti-inflammatory activity by decreasing C-reactive protein serum levels [86].

Apoptosis may be triggered extrinsically by the activation of the Fas receptor, or intrinsically through a process modulated by the BCL-2 family of proteins [87]. BAX also constitutes a modulator of the intrinsic apoptotic pathway, leading to mitochondrial membrane permeabilization. This process determines the release of cytochrome-c, triggering apoptosis through caspase-9 [88]. The decreased levels of BLC-2, BAX, as well as Caspase-9 in ascites fluid samples following the three therapeutic approaches, may signify the cessation of the apoptosis process. The activation of caspase-9 causes the cleaving and activation of caspase-3, leading once again to cellular death [87]. The decreased levels of caspase-3 (although lacking statistical significance) were also suggestive of the lowered apoptosis rate. These findings were in accordance with an in vitro study on hormone-dependent prostate cancer cells. The proposed cause of this biological modification was also the activity of the compound oenothein B [59]. p53 constitutes a tumor suppressor gene while also being responsible for apoptosis, limiting cellular proliferation. p53 activation leads to BAX activation, cytochrome-c release, and caspase-9 activation [89]. The reduced levels of p53 expression may also indicate an antiproliferative effect of the EH extract. An in vitro study performed on HT-29 human colorectal carcinoma cells demonstrated the increase in mRNA expression levels for apoptotic genes such as caspase-3, caspase-8, Bax, and the reduction in the p53 gene expression after exposure to aqueous and ethanolic extracts of *Epilobium parviflorum* Schreb. This indicated the presence of apoptosis in the tested cancerous cells, thus negatively contributing to their proliferation [88]. 

The hepatoprotective action of the extract was noticed against tumoral cell metastasis, and a prominent action related to Cph side effects such as hepatocytes degeneration or necrosis was not found. These assumptions were derived from an integrative view of the histopathological exams (H&E staining) and biochemical markers (MDA, CAT, GSH, GSSG, GPX1) of the liver samples. The exposure to CPh and then the extract administration were related to lowering oxidative damages by non-enzyme-based antioxidant response as a main reaction pathway via GSH and GSSG, and, secondly, the catalase was involved to sustain the redox balance and cell integrity. In addition, the extract administration restored the hepatic reticulin frame, whereas Cph and other treatment combinations were not associated with reticulin restoration, which was associated with malignant status and/or liver failure according to the findings of Putra et al. [90]. Van Gieson and Mallory staining techniques did not reveal any fibrotic alterations, corroborating the necrosis and degenerative impact attributed to Cph treatment. Simultaneously, these staining results support the antimetastatic and liver-protective properties of the optimized EH extract. This aligns with the pharmacological observations made by Dzhafar et al. [84], who similarly investigated the therapeutic properties of *E. hirsutum* extract. The findings from our study have suggested an increased liver hemodynamic plasticity based on the normal distribution of the perivascular reticulin and an improvement in nutrients’ transition from blood to hepatocytes as one of the main aspects of the protective pathways associated with *E. hirsutum*.

These results, which corroborated recent findings, suggest that the bioactive potency of EH is attributed to its diverse phytocomplex. Compounds such as oenothein B, caftaric acid, hyperoside, quercitrin, myricetin, kaempferol, gallic acid, beta-sitosterol, and tocopherols, among others, all quantified in previous studies, have demonstrated significant antioxidant, anti-inflammatory, and antitumor effects in vitro across normal and cancerous cell lines [26,27,34]. The synergistic effects of these phytoconstituents within the EH optimized extract are crucial in modulating inflammatory pathways, mitigating oxidative stress, and influencing tumor cell viability. This holistic approach underscores the importance of evaluating the collective impact of these compounds, offering a more comprehensive understanding of EH’s therapeutic potential.

## 4. Materials and Methods

### 4.1. Chemical and Reagents

Indomethacin, carboxymethyl cellulose, and Lambda carrageenan type IV were purchased from Sigma-Aldrich (Taufkirchen, Germany). 2-thiobarbituric acid and Bradford reagent were acquired from Merck KGaA (Darmstadt, Germany) and ELISA tests for cytokines (TNF-α, IL-6) were purchased from Elabscience (Houston, TX, USA). All HPLC reagents and standards were of analytical grade and were acquired from Sigma-Aldrich (Germany).

### 4.2. Plant Material and Extract Preparation

The plant material, *Epilobium hirsutum* L. aerial parts (*Epilobii hirsutii herba*), was harvested from wild population from Suceava county, Romania, during the flowering stage (47.6327, 26.2476). The plant species was authenticated by botany Professor Gianina Crișan from the Department of Pharmaceutical Botany, Faculty of Pharmacy, Iuliu Hatieganu University of Medicine and Pharmacy, Cluj-Napoca. A voucher specimen was deposited in the herbarium of this department [27].

The plant material was washed with tap water to remove any remaining impurities, and then it was air-dried for 5 days, safe from sunlight, at room temperature (25 °C).

To obtain the optimized extract rich in polyphenols, a design of the experiments was employed by using Modde software 13.2.0 (Sartorius Stedim Biotech GmbH, Göttingen, Germany). A detailed presentation of the experimental design, and the screening and optimization steps are provided in a previously published paper [27]. The optimized extract was obtained via ultra-turrax assisted extraction technique (4000 rpm), for 8 min, in hydroalcoholic mixture of solvents with 30% ethanol. After extraction, the ethanol was removed by using a rotavapor and the water was removed through further lyophilization. The lyophilizate was kept at −20 °C prior to animal testing [27]. 

### 4.3. Phytochemical Characterization of Optimized Extract

The complete phytochemical profile included the quantitative determination of compounds from various chemical classes such as polyphenol carboxylic acids, flavonoids, sterols, tocopherols, and the most abundant bioactive compound, oenothein B, which is an ellagitannin. The details about the used equipment, chromatographic column, mobile phase, elution conditions, and determined concentrations for each bioactive compound can be found in a previously published paper, along with the UV chromatogram and TIC in the supplementary materials [27].

### 4.4. Study Protocol for Animal Testing

The animal study protocol was approved by the Institutional Animal Ethical Committee (IAEC) of the Iuliu Hațieganu University of Medicine and Pharmacy Cluj-Napoca, Romania, and by the National Sanitary Veterinary and Food Safety Agency from Romania (no. 289/09.02.2022). The study protocol was written in accordance with Directive 2010/63/EU on the protection of animals used for scientific purposes and guidelines for Animal Welfare. 

#### 4.4.1. Acute Rat Paw Inflammation

The in vivo assessment was conducted using a male Wistar rat model. The animals included in this study had a weight ranging from 190 to 240 g and were procured from the animal department of Iuliu Hatieganu University of Medicine and Pharmacy, Cluj-Napoca. The rats were further acclimated in the Physiology Department’s vivarium under specific conditions, including 12 h light and dark cycles, 35% humidity, unrestricted access to water, and a standard normocaloric diet (VRF1). The rats were randomized into three groups, each consisting of eight specimens. Over a span of four days, treatment was administered through oral gavage, with a maximum volume of 0.6 mL, as follows: Group 1 received a 2% carboxymethyl cellulose solution (CMC) (negative control); Group 2 received 5 mg of Indomethacin per kg of body weight (b.w.) in a 1.5% carboxymethyl cellulose solution (IND) (positive control); and Group 3 received 10 mg of oenothein B per 100 g of b.w. from the optimized *E. hirsutum* extract (EH).

On the fifth day, inflammation was induced by injecting 100 μL of freshly prepared 1% carrageenan (λ-carrageenan, type IV, Sigma-Aldrich, Taufkirchen, Germany) diluted in normal saline into the right hind footpad. An equivalent volume of saline solution was injected into the left hind paw, serving as a negative control. Subsequently, paw samples were collected at 2 and 24 h after the carrageenan injection. These samples were obtained under general anesthesia induced by intraperitoneal injection of 90 mg/kg of ketamine and 10 mg/kg of xylazine. After homogenization in a pH 7.4 buffer containing 50 mM TRIS and 10 mM EDTA, the collected samples were evaluated for oxidative stress parameters and cytokine levels. The protein content was determined using the Bradford method [91].

The rat paw volume was assessed using a plethysmometer (model 37140, UGO Basile, Comerio, VA, Italy). The paws measurements were performed before and after carrageenan injection at 2 and 24 h. The modification in rat paw volume was determined with the following formula: Dv (mL) (%) = [(Vi (mL) − Vb (mL))/Vd (mL)] × 100. Dv represents the difference between the paws’ volumes, Vi is the inflamed paw volume after carrageenan administration, and Vb is the basal volume of the same paw before the carrageenan injection.

#### 4.4.2. Ehrlich Ascites Carcinoma

For the in vivo assessment of the antitumor activity of *E. hirsutum* optimized extract, forty 8-week-old Swiss albino male mice with Ehrlich ascites carcinoma (EAC), each weighing approximately 30 g, were included in the study. These mice were procured from the animal department of Iuliu Hatieganu University of Medicine and Pharmacy, Cluj-Napoca, and allowed 24 h to acclimate in the Physiology Department’s vivarium.

The mice were then randomly divided into four groups, each consisting of ten mice. A volume of 1 mL of ascites liquid containing 1 million cells from a donor animal was inoculated into each mouse. After 24 h, the mouse commenced the experiment and were treated orally for ten days as follows:

- Group 1 received 0.25 mL of 2% carboxymethyl cellulose (CMC), serving as the negative control (A).
- Group 2 received 25 mg per kg of b.w. of cyclophosphamide (Cph), acting as the positive control.
- Group 3 received 25 mg per kg of b.w. of cyclophosphamide (Cph) with 10 mg of oenothein B per 100 g of b.w. from the optimized extract *E. hirsutum* (Cph + EH).
- Group 4 received 10 mg of oenothein B per 100 g of b.w. from the optimized extract *E. hirsutum* (EH).

Throughout the ten-day period, the animals were closely monitored. After the final treatment, under general anesthesia induced by intraperitoneal injection of 90 mg/kg ketamine and 10 mg/kg xylazine, ascites fluid, liver, and heart tissue were collected. These samples were preserved at −80 °C until further analysis. 

### 4.5. Oxidative Stress Evaluation

Oxidative stress parameters were assessed in plantar tissue homogenates and in ascites samples, liver, and heart homogenates through the quantification of malondialdehyde (MDA) through a spectrofluorimetric assay using 2-thiobarbituric acid method. Moreover, all samples underwent assessment for levels of reduced (GSH) and oxidized (GSSG) glutathione, including the calculation of their ratio (GSH/GSSG). The activities of catalase (CAT) and glutathione peroxidase (GPx) were also measured.

Liver fragments were collected and homogenized using a polytron homogenizer (Brinkman Kinematica, Switzerland) followed by the preparation of a cytosolic fraction for oxidative stress assessment, as previously described [92]. Protein levels in liver tissue homogenates and ascites samples were quantified using the Bradford method [93].

### 4.6. Proinflammatory Cytokine Investigation 

TNF-α and IL-6 levels in plantar tissue homogenates, ascites fluid, liver, and heart homogenates were assessed through ELISA assays following the manufacturer’s protocol, with results expressed in pg/mg protein.

### 4.7. Western Blot Analysis

Lysates (20 µg protein/lane) underwent electrophoresis on SDS PAGE gels, followed by transfer to polyvinylidene difluoride membranes using the Biorad Miniprotean system (Bio-Rad Laboratories, Hercules, CA, USA). The blots were blocked and then incubated with antibodies against p53, BCL-2, BAX, COX2, NFκB, pNFκB, and NRF2 (Santa Cruz Biotechnology, Santa Cruz, CA, USA), as well as caspase-3 and caspase-9 (Antibodies Online, Atlanta, GA, USA). Subsequently, the blots were washed and exposed to corresponding secondary peroxidase-linked antibodies (Santa Cruz Biotechnology, Santa Cruz, CA, USA). Protein detection was performed using Supersignal West FemtoChemiluminiscent substrate (Thermo Fisher Scientific, Rockford, IL, USA), and analysis was conducted using a Gel Doc Imaging system equipped with a XRS camera and Quantity One^®^ 1-D analysis software 4.6 (Bio-Rad Laboratories, Hercules, CA, USA). Glyceraldehyde 3-phosphate dehydrogenase (GAPDH) acquired from Santa Cruz Biotechnology (Santa Cruz, CA, USA) served as a protein-loading control.

### 4.8. Histopathological Analysis

For the evaluation of histological features, the biopsies were isolated and fixed in a 5% neutral formalin solution for 48 h. After paraffin embedding, sections were cut at 5 µm and mounted on electrostatically charged glass slides. Tissue sections were dewaxed in xylene, rehydrated, and stained with hematoxylin–eosin, the Mallory procedure, and the reticulin and Van Gieson methods for the multifaceted histological exam. Then, the slides were blindly investigated by a histologist using an Optika trinocular microscope B383-FL with MDC CCD Camera 2 MP (Optika Microscopes, Ponteranica, BG, Italy). The histological changes (reticulin deposits and collagen infiltration) were semi-quantitatively evaluated following the method of Grover et al. [94] and Toma et al. [95]. 

### 4.9. Multivariate Data Analysis 

Orthogonal Projections to Latent Structures based Discriminant Analysis (OPLS-DA) was applied to investigate the treatment-induced differences in the expression profiles of the selected variables/biological parameters with respect to the negative control treatment. Before fitting the models, the X dataset of the model, represented by the expression data, and the Y dataset, represented by a (0/1) dummy variable matrix assigning class membership, were scaled to unit variance. The performance of the OPLS-DA models was evaluated through the percentage of explained variability (R^2^X, R^2^Y) and the predictive capacity (Q^2^) calculated through a cross-validation procedure. 

For an easy visualization of the differences and similarities between treatments, shared and unique structures (SUS) graphs were represented by plotting the modeled correlation vector (p corr) of two separate OPLS-DA models. P (corr vector) reflects the reliability of treatment-induced changes in the expression of biological parameters and expresses the correlation between each X variable and t score vector as values from −1 to 1. 

**Figure 18:**
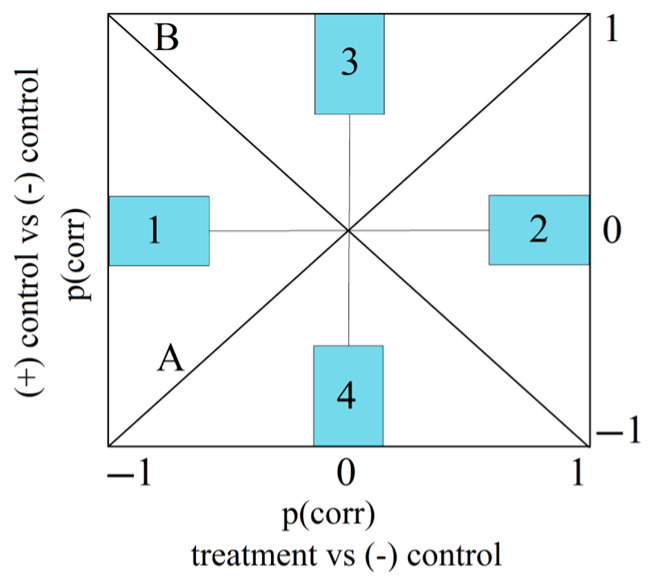
SUS plot interpretation. Shared effects: Diagonal A—in the same direction; Diagonal B—in opposite direction. Unique effects: Region 1—unique decrease—and Region 2—unique increase induced by “treatment”; Region 3—unique increase—and Region 4—unique decrease induced by (+) control.

The vertical axis of these plots always represents the correlation vector of the OPLS-DA model that relates to the comparison of the positive control versus negative control treatment (Figure 18). The horizontal axis refers to the correlation vector of the OPLS-DA model that compares the plant extract or the co-administration of positive control and plant extract with the negative control treatment. The shared effects are identified by searching for variables found on two imaginary diagonals drawn on the plots. Variables displayed on diagonal (A) are influenced in the same direction, whereas variables displayed on diagonal (B) are influenced in an opposite manner by the two treatments. Unique effects, or biological parameters influenced by only one treatment, are positioned near the horizontal and vertical axes and have a high reliability through an increased absolute value of its projection onto one of the axes [96,97].

### 4.10. Statistical Analysis

In the statistical analysis of the data obtained from preclinical animal studies, a Shapiro–Wilk test was initially conducted, suitable for analyzing small sample sizes such as the study groups consisting of 8 or 10 experimental animals. This test confirmed the normal distribution of the data, thereby validating the use of one-way ANOVA and Tukey’s multiple comparisons post-hoc test for further statistical evaluation. These analyses were performed using GraphPad Prism, version 9.3.0 (GraphPad Software, Boston, MA, USA). Statistical significance was determined for *p* values below 0.05, and the results were expressed as mean values ± standard deviation (SD). 

## 5. Conclusions

A detailed investigation of the optimized *Epilobium hirsutum* (EH) extract has unveiled its substantial potential as both an anti-inflammatory and antioxidant agent. This extract demonstrated significant anti-inflammatory effects in a rat model of acute paw inflammation, affirming its therapeutic benefits in managing acute inflammatory responses. Moreover, it exhibited antioxidant and anti-inflammatory properties in a murine model of Ehrlich ascites carcinoma. These findings indicate that EH’s bioactive compounds, including oenothein B, caftaric acid, hyperoside, quercitrin, myricetin, kaempferol, gallic acid, beta-sitosterol, and tocopherols, can modulate inflammatory pathways, reducing oxidative stress markers and influencing tumor cell viability. Importantly, the data suggest that the EH extract could enhance the efficacy and mitigate the side effects of conventional chemotherapy, such as cyclophosphamide. This synergistic effect is likely due to EH’s phytoconstituents, offering a multifaceted approach to modulating the tumor microenvironment and the host’s responses, especially considering that cancer-causing mutations may accumulate due to chronic inflammation and persistent oxidative stress. Future research examining various Cph/EH ratios could provide deeper insights into their synergistic potential and assist in optimizing cancer treatment strategies.

The encouraging outcomes from these animal studies pave the way for future research to elucidate the molecular mechanisms of the EH extract, refine dosing strategies, and conduct extensive clinical trials. These steps are crucial to establish the EH extract’s role in cancer therapy, potentially leading to more efficacious and less toxic treatment methods. Further studies might explore additional inflammation markers like prostaglandin E2 (PGE2), nitric oxide (NO), interleukin-1 beta (IL-1β), and tumor progression through flow cytometry, angiogenesis markers such as vascular endothelial growth factor (VEGF), and apoptosis-related markers including caspase-8, cytochrome C release, and PARP cleavage. Hence, the optimized EH extract could serve as a valuable complementary therapy in oncology and inflammatory diseases, and aid in the recovery post chemotherapy.

## Figures and Tables

**Table 1:** The histological changes evaluated semi-quantitatively (reticulin deposits and collagen infiltration).

	Group 1 Ascites	Group 2 Ascites + Cyclophosphamide	Group 3 Cyclophosphamide + *E. hirsutum* Extract	Group 4 *E. hirsutum* Extract
Reticulin	0	4+	2+	1+
Van Gieson	0	0	3+	1+

where 0 is negative reaction, 1+ represents low reaction (20–40%), 2+ indicates moderate reaction (40–60%), 3+ signifies intense reaction (60–80%), and 4+ indicates very intense reaction (80–100%).

## Data Availability

The information concerning the complete phytochemical profile of the *Epilobium hirsutum* optimized extract can be found at the following link, including https://www.mdpi.com/2076-3921/12/1/91 and supplementary materials (can be downloaded at https://www.mdpi.com/article/10.3390/antiox12010091/s1). Otherwise, the data are contained within the article.
